# Supplementary material for: IL-6 regulates autophagy and chemotherapy resistance by promoting BECN1 phosphorylation
Source: Nat Commun. 2021 Jun 15;12:3651. doi: 10.1038/s41467-021-23923-1 (PMC8206314; doi:10.1038/s41467-021-23923-1)
Supplement: Supplementary file 1 — Supplementary Information [file 41467_2021_23923_MOESM1_ESM.pdf]

Supplementary information

IL-6 regulates autophagy and chemotherapy resistance by promoting BECN1 phosphorylation

Fuqing Hu [1&], Da Song[1&], Yumeng Yan[2], Changsheng Huang[1], Jingqin Lan[1], Yaqi Chen[1], Anyi Liu[1], Qi Wu[1], Li Sun[3], Feng Xu[1], Fayong Hu[1], Lisheng Chen[1], Xuelai Luo[1], Yongdong Feng[1], Shengyou Huang[2], Junbo Hu[1#] and Guihua Wang[1#]

1, GI Cancer Research Institute, Tongji Hospital, Huazhong University of Science and Technology, Wuhan, 430030, P. R. China.

2, School of Physics, Huazhong University of Science and Technology, Wuhan, Hubei 430074, P. R. China.

3, Department of Oncology, Tongji Hospital, Huazhong University of Science and Technology, Wuhan, 430030, P. R. China.

Supplementary Fig. 1

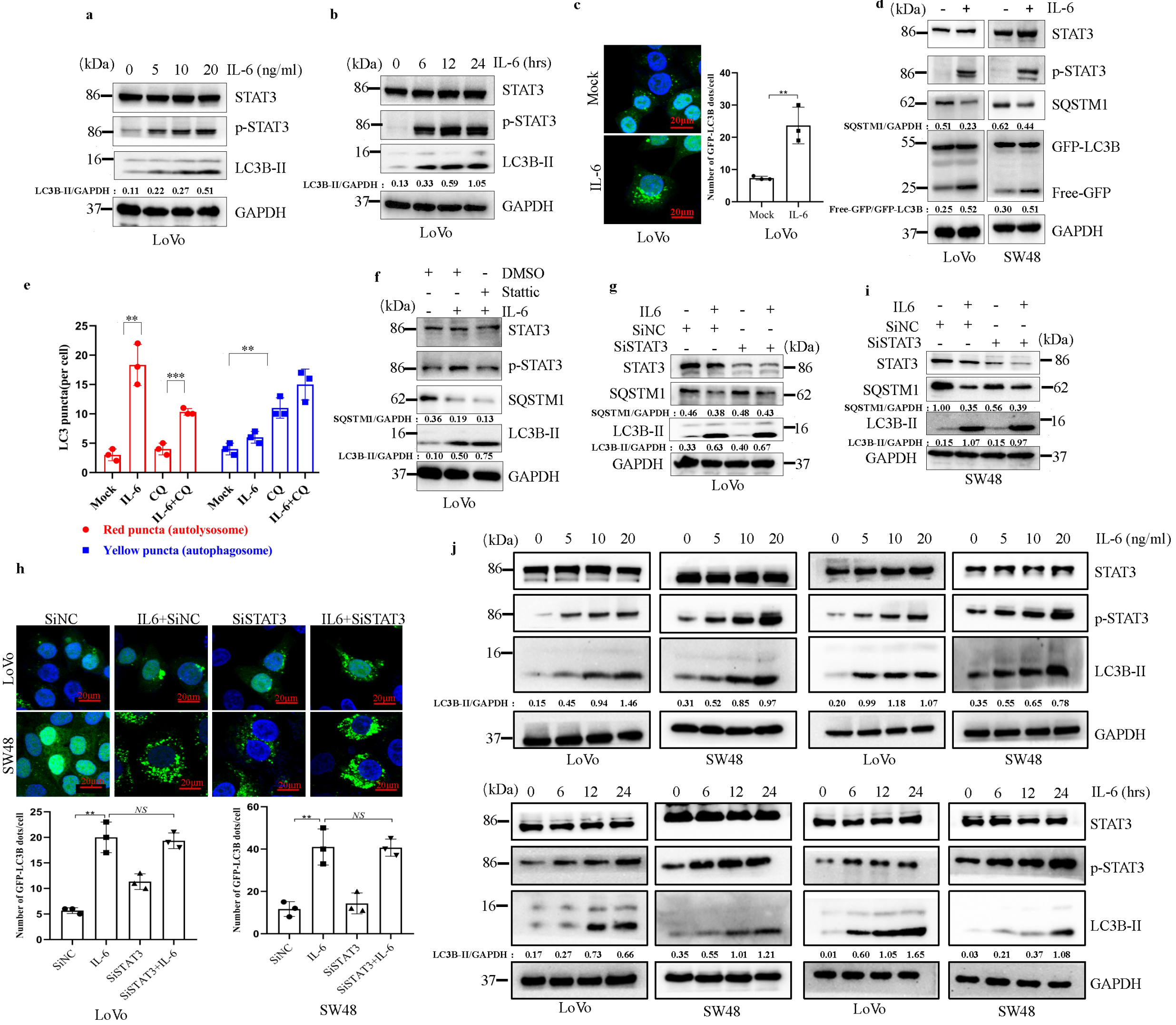

Supplementary Fig. 1 IL-6 triggers autophagy in cells via a STAT3-independent pathway. (a-b) Exogenous IL-6 promotes the accumulation of LC3B-II in LoVo cells treated with IL-6 in a dose- (a) and time-dependent (b) manner. Western blotting was performed to examine the expression of LC3B-II and SQSTM1. **(c)** Exogenous IL-6 (20 ng/ml) promotes GFP-LC3B puncta in LoVo cells. Quantitative analysis of GFP-LC3B puncta is shown in the right panel (n = 3 independent experiments). **P<0.01. Scale bars, 20 μm. **(d)** Exogenous IL-6 (20 ng/ml) promotes the expression of free GFP and the degradation of SQSTM1 in LoVo cells expressing the GFP-LC3B fusion protein. Western blotting was performed to examine the levels of free GFP and SQSTM1 (n = 2 independent experiments). **(e)** Quantification of autophagic flux with the mCherry-GFP-LC3 reporter in SW48 cells stably expressing the mCherry-GFP-LC3B fusion protein and separately treated with IL-6 (20 ng/ml) in the absence or presence of CQ treatment. The number of yellow or red puncta in each cell was calculated (n = 3 independent experiments) **P<0.01, ***P<0.001. **(f)** Western blotting was performed for LoVo cells separately treated with IL-6 (20 ng/ml) in the absence or presence of Stattic (20 μM) (n = 2 independent experiments). **(g-i)** SW48 (g) and LoVo (h) cells were separately transfected with small interfering RNA targeting STAT3 (SiSTAT3) and small interfering RNA targeting a negative control gene (SiNC). After transfection for 24 hours and following stimulation with IL-6 (20 ng/ml) for 24 hours, Western blotting was performed to examine the expression of LC3B-II and SQSTM1 (n = 2 independent experiments). **(h)** SW48 and LoVo cells were separately transfected with small interfering RNA targeting STAT3 (SiSTAT3) or small interfering RNA targeting a negative control gene (SiNC). After transfection for 24 hours, and following stimulation with IL-6 (20 ng/ml) for 24 hours, confocal microscopy images were obtained. Quantitative analysis of GFP-LC3B puncta is shown in the lower panel (n = 3 independent experiments). **P<0.01, NS, Not significant. Scale bars, 20 μm. (**j**) Exogenous IL-6 promotes LC3B-II accumulation in LoVo and SW48 cells treated with IL-6 in a dose- and time-dependent manner. Western blotting was performed to examine the expression of LC3B-II and SQSTM1 (n = 3 independent experiments). In (c, e and h), the values are presented as the means ± s.d.; p values (Student's t-test, two-sided) with comparisons made to the control or different indicated groups are shown. Source data are provided in the Source Data file.

Supplementary Fig. 2

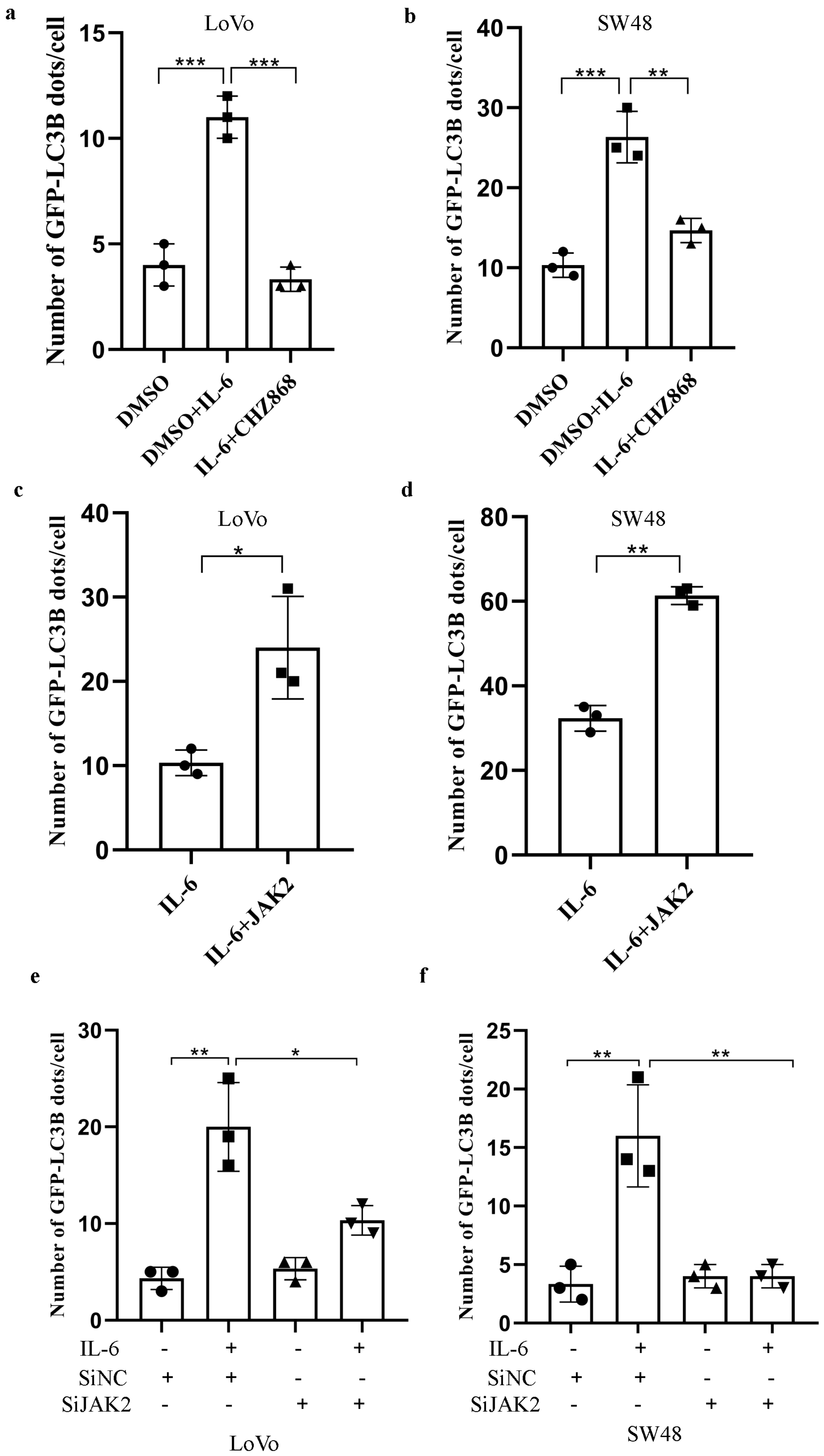

Supplementary Fig. 2 IL-6 promotes autophagy in a JAK2 signaling-dependent manner. (a-b) Quantification of GFP-LC3B puncta in LoVo (a) and SW48 (b) cells, $^{**}P<0.01$, $^{***}P<0.001$. (c-d) Quantification of GFP-LC3B puncta in LoVo (c) and SW48 (d) cells, $^{*}P<0.05$, $^{**}P<0.01$. (e-f) Quantification of GFP-LC3B puncta in LoVo (e) and SW48 (f) cells, $^{*}P<0.05$, $^{**}P<0.01$. The results were obtained from three independent experiments. In (a, b, c, d, e and f), the values are presented as the means $\pm$ s.d.; p values (Student's t- test, two-sided) with comparisons made to the control or different indicated groups shown. Source data are provided in the Source Data file.

Supplementary Fig. 3

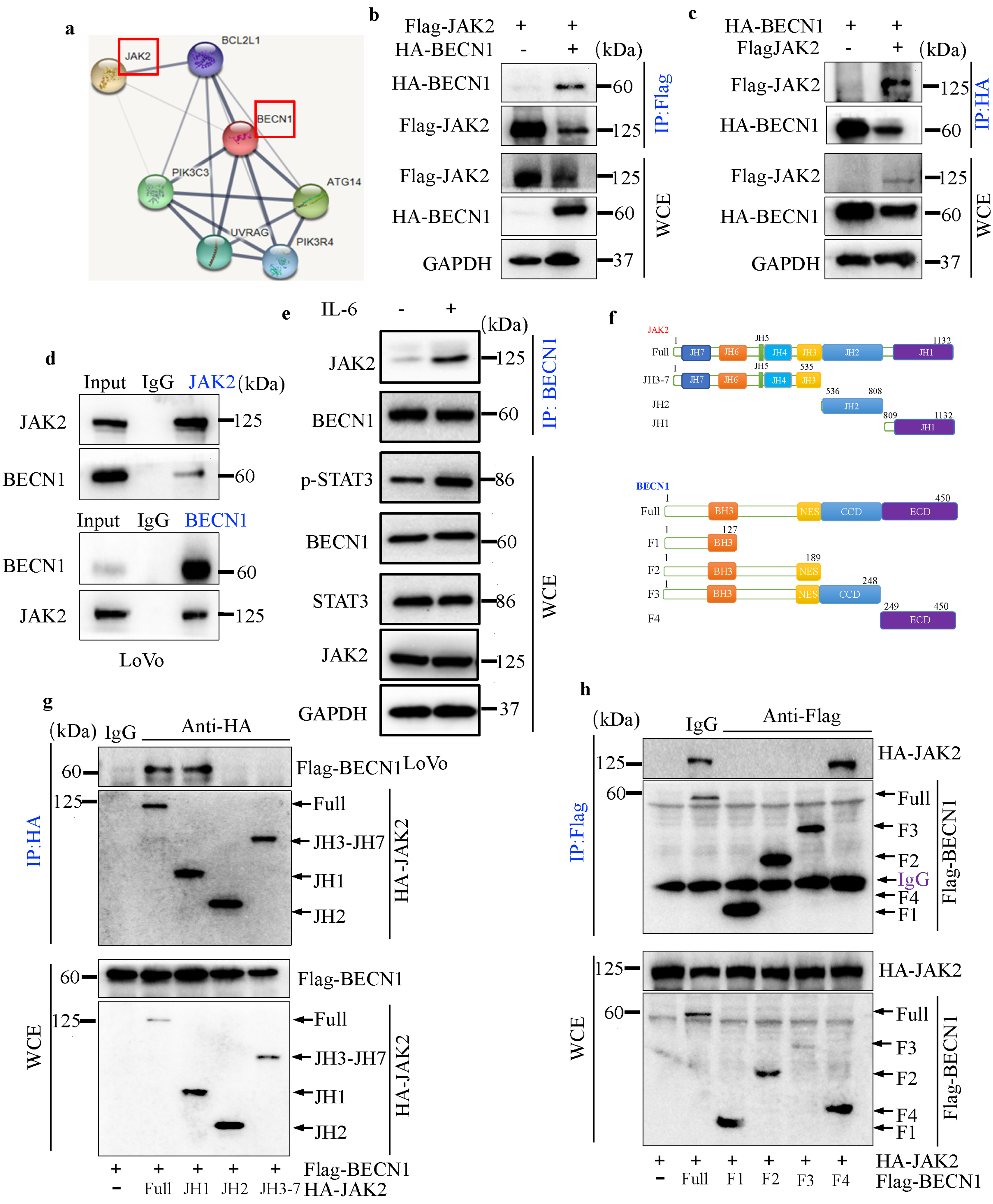

Supplementary Fig. 3 IL-6 stimulates the interaction between JAK2 and BECN1. (a) Prediction of protein interactions in the STRING database showing two partner proteins, JAK2 and BECN1. (b) IP analyses for HA-BECN1 and Flag-JAK2 in HEK293T cells expressing Flag-JAK2 or Flag-JAK2 and HA-BECN1. Western blotting was performed on WCEs (lower panels) (n = 2 independent experiments). (c) IP analyses for Flag-JAK2 and HA-JAK2 in HEK293T cells expressing HA-BECN1 or Flag-JAK2 and HA-BECN1. Western blotting was performed on WCEs (lower panels) (n = 2 independent experiments). (d) IP analyses were performed to examine the interaction between BECN1 and JAK2 (top) or JAK2 and BECN1 (bottom) in LoVo cells. (n = 2 independent experiments). (e) Co-IP was performed to examine the relationship between JAK2 and BECN1 in LoVo cells in the absence or presence of IL-6 (20 ng/ml) for 12 hours (upper panels). Western blotting was performed on WCEs (lower panels) (n = 2 independent experiments) (f) Schematic representation of N-terminal HA-tagged full-length JAK2 (full), along with the indicated deletion mutants (upper panels). Schematic representation of N-terminal Flag-tagged full-length BECN1 (full), along with the indicated deletion mutants (lower panels). (g) IP analyses of HA-JAK2 and Flag-BECN1 in HEK293T cells expressing Flag-BECN1, HA-JAK2 (full) and HA-JAK2 mutants (JH1, JH2 and JH3-JH7). Western blotting was performed on WCEs (lower panels) (n = 2 independent experiments). (h) IP analyses of HA-JAK2 and Flag-BECN1 in HEK293T cells expressing HA-JAK2, Flag-BECN1 (full) and Flag-BECN1 mutants (F1, F2, F3 and F4). Western blotting was performed on WCEs (lower panels) (n = 2 independent experiments). WCE = whole cell extract. Source data are provided in the Source Data file.

Supplementary Fig. 4

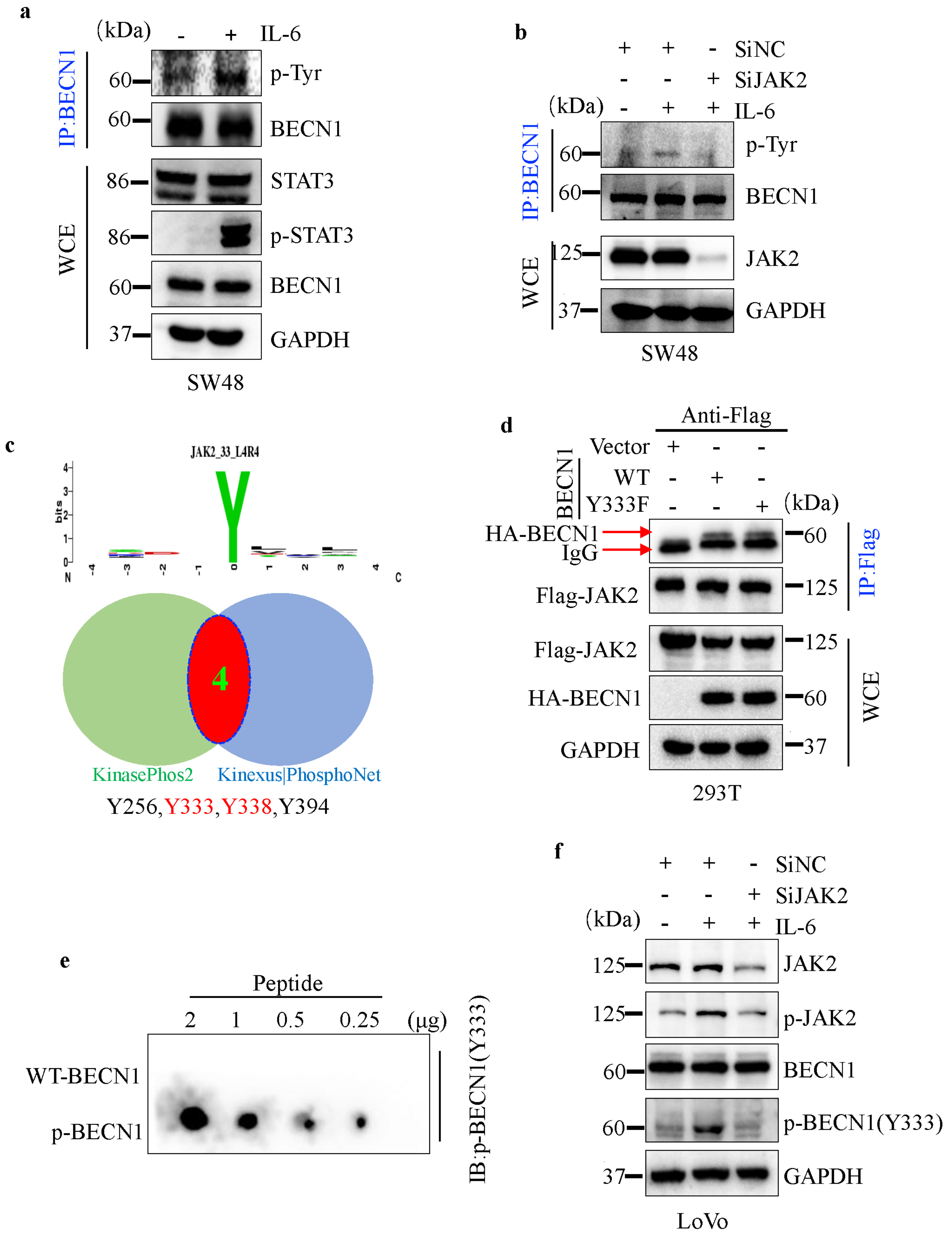

Supplementary Fig. 4 IL-6 promotes the phosphorylation of BECN1 at Y333. (a) Co-IP analyses for p-Tyr and BECN1 in SW48 cells in the absence or presence of IL-6 (20 ng/ml) for 12 hours. Western blotting was performed on WCEs (lower panels) (n = 2 independent experiments). (b) Co-IP analyses for p-Tyr and BECN1 in SW48 cells separately transfected with SiJAK2 and SiNC in the absence or presence of IL-6 (20 ng/ml) for 12 hours. Western blotting was performed on WCEs (lower panels) (n = 2 independent experiments). (c) Prediction of the site in BECN1 phosphorylated by JAK2 through a multibiodatabase analysis. (d) IP analyses for HA-BECN1 and Flag-JAK2 in HEK293T cells separately transfected with vector, Flag-JAK2, HA-BECN1-WT or HA-BECN1-Y333F. Western blotting was performed on WCEs (lower panels) (n = 2 independent experiments). (e) In vitro dot blot analysis of p-BECN1 (Y333) for the peptide containing phosphorylated Y333. (f) LoVo cells were separately transfected with SiJAK2 and SiNC. After transfection for 48 hours and following stimulation with IL-6 (20 ng/ml) for 12 hours, Western blotting was performed (n = 2 independent experiments). WCE = whole cell extract. Source data are provided in the Source Data file.

Supplementary Fig. 5

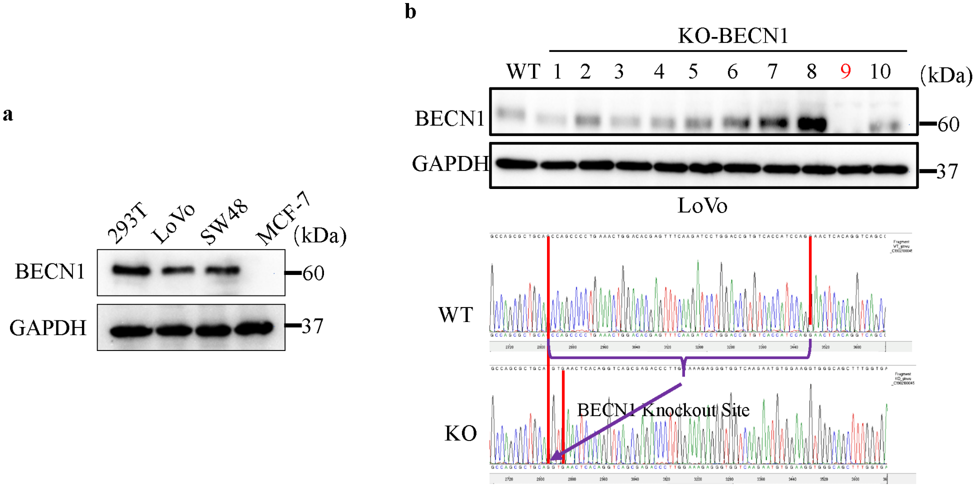

Supplementary Fig. 5 Deletion of endogenous BECN1 in MCF7 and LoVo cell lines. (a) Western blotting was performed to examine the expression of endogenous BECN1 in different cell lines. (b) Western blot analyses of the effects of BECN1 KO using gRNAs on BECN1 in LoVo cells. Genomic DNA sequencing analysis of LoVo-BECN1-KO cells. The sequencing results showed the generation of BECN1 KO cell lines with deletion mutations (lower panel). Source data are provided in the Source Data file.

Supplementary Fig. 6

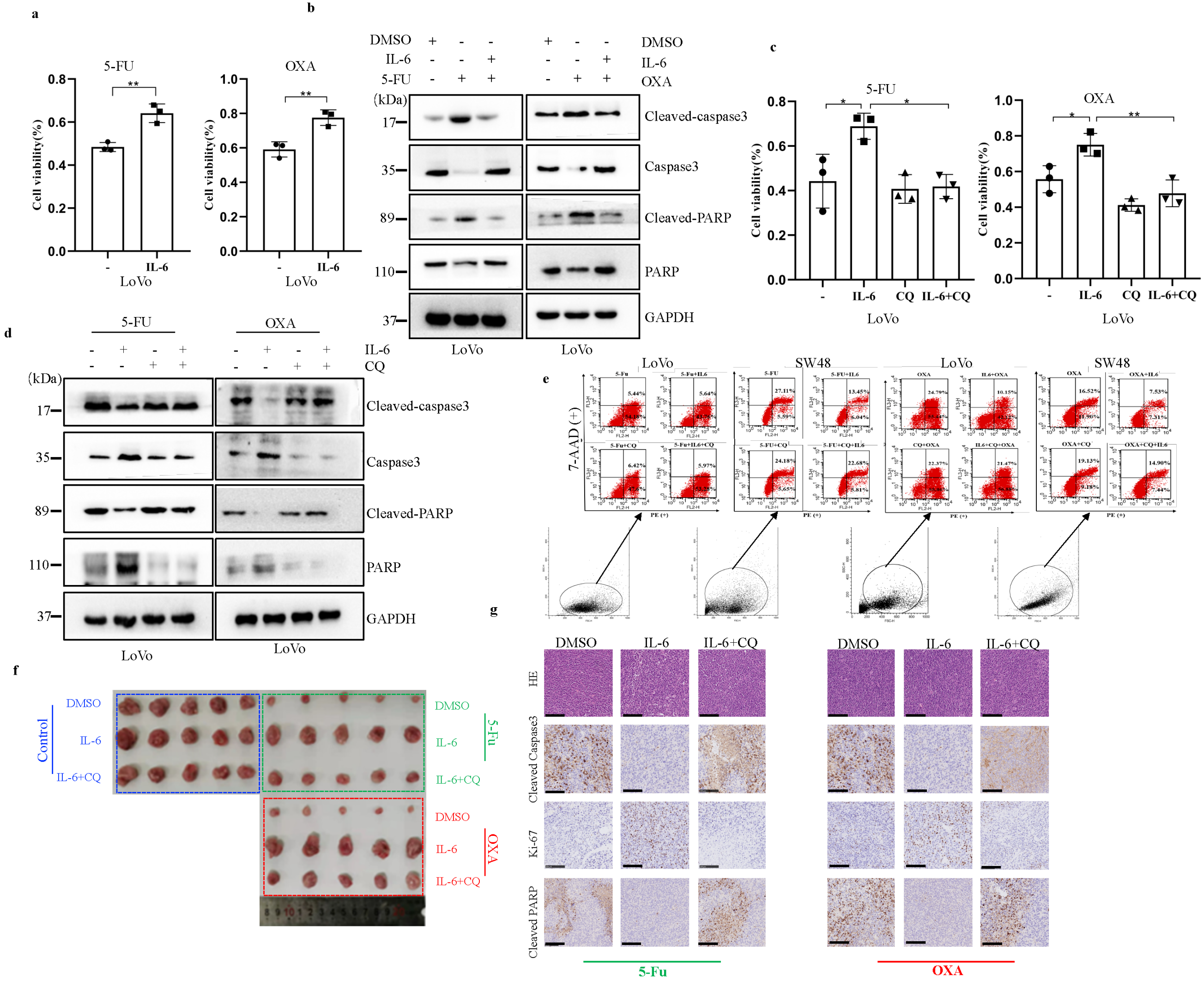

Supplementary Fig. 6 Inhibition of IL-6-induced autophagy enhances chemotherapy sensitivity. (a) CCK-8 assays for cell viability of LoVo cells separately pretreated with DMEM or IL-6 (20 ng/ml) for 8 hours in the presence of DMSO, 5-Fu (800 μM) or OXA (100 μM) for 36 hours. **P<0.01 (n = 3 independent experiments). (b) Western blot analyses for cleaved caspase-3 and cleaved PARP1 in LoVo cells separately pretreated with DMEM or IL-6 (20 ng/ml) for 8 hours in the presence of DMSO, 5-Fu (800 μM) or OXA (100 μM) for 36 hours (n = 2 independent experiments). (c) CCK-8 assays for cell viability of LoVo cells separately pretreated with DMEM or IL-6 (20 ng/ml) for 8 hours in the presence of CQ (25 μM), 5-Fu (800 μM) or OXA (100 μM) for 36 hours. *P<0.05, **P<0.01 (n = 3 independent experiments). (d) Western blot analyses for cleaved caspase3 and cleaved PARP1 in LoVo cells separately pretreated with DMEM or IL-6 (20 ng/ml) for 8 hours in the presence of CQ (25 μM), 5-Fu (800 μM) or OXA (100 μM) for 36 hours (n = 2 independent experiments). (e) Apoptosis was assessed by flow cytometry of SW48 and LoVo cells separately pretreated with DMEM or IL-6 (20 ng/ml) for 8 hours in the presence of CQ (25 μM), 5-Fu (800 μM) or OXA (100 μM) for 36 hours. Gating strategy to identify apoptotic cells with different treatments. Relative percentages for the gate are presented on the contour plot below, while event counts for each quadrant are presented on the four scatter plots above. (f) In vivo CRC xenografts derived from CT26 cells were treated with IL-6, CQ, 5-Fu or OXA. Representative data of tumors in mice bearing CT26 cells in different groups. (g) Representative results of immunohistochemical staining for cleaved caspase3, cleaved PARP1 and Ki-67 in tumor tissue from different CRC xenografts. Scale bars, 20 μm. In (a and c), the values are presented as the means ± SEM; p values (Student's t-test, two-sided) with control or different indicated groups are shown. Source data are provided in the Source Data file.

Supplementary Fig. 7

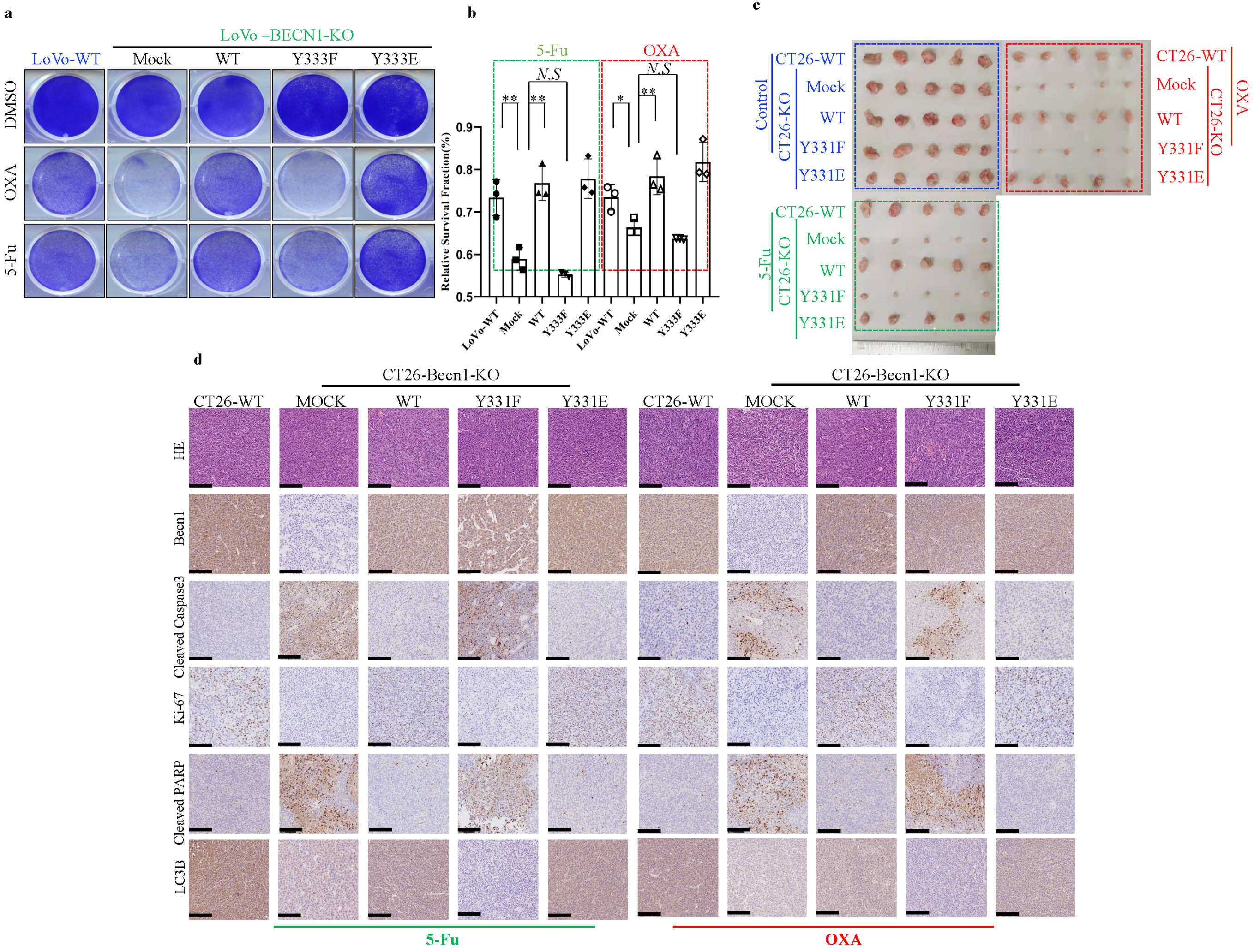

Supplementary Fig. 7 Phosphorylation of Y333 in BECN1 is necessary for CRC chemotherapy resistance. (a-b) Colony formation analyses of LoVo and LoVo-BECN1-KO cells separately expressing a vector control (Mock), HA-BECN1 WT, HA-BECN1 Y333F or HA-BECN1 Y333E and treated with DMSO, 5-Fu (800 μM) or OXA (100 μM) for 28 days (a). Quantification of the relative survival fraction is shown (b). (n = 3 independent experiments) *P<0.05, **P<0.01, ***P<0.001, NS, Not significant. (c) In vivo CRC xenografts derived from CT26 cells and CT26-BECN1-KO cells separately expressing a vector control (Vector), BECN1 WT, BECN1 Y331F or BECN1 Y331E and treated with DMSO, 5-Fu or OXA. Representative data of tumors in mice bearing CT26 cells in different groups. (d) Representative results of immunohistochemical staining for BECN1, cleaved caspase3, cleaved PARP1, LC3B and Ki-67 in tumor tissues from different CRC xenografts. Scale bars, 20 μm (n = 5 mice per genotype). In (b), the values are presented as the means ± SEM; p values (Student's t-test, two-sided) with comparisons made to the control or different indicated groups are shown. Source data are provided in the Source Data file.

Supplementary Table 1. Primers for plasmid construction

Gene	Sequences
Human BECN1(WT/Full)	F: ATGGAAGGGTCTAAGACGTCC<br>R: TCATTTGTTATAAAATTGTGA
Human BECN1(Y333F)	F: TACCGACTTGTTCCTTTCGGAAACCAT<br>R: AAAGGAACAAGTCGGTATCTCTGAAATT
Human BECN1(Y333E)	F: TACCGACTTGTTCCTGAAGGAAACCATTCA<br>R: TTCAGGAACAAGTCGGTATCTCTGAAATT
Human BECN1(F1)	F: ATGGAAGGGTCTAAGACGTCC<br>R: CGACATGATGTCAAAAAGGTCC
Human BECN1(F2)	F: ATGGAAGGGTCTAAGACGTCC<br>R: TAGTGCCAGCTCCTTTAGCTCCAT
Human BECN1(F3)	F: ATGGAAGGGTCTAAGACGTCC<br>R: CTTCAGCTCATCATCCAGCTCCA
Human BECN1(F4)	F: AAGAGTGTTGAAAACCAGATGCG<br>R: TCATTTGTTATAAAATTGTGA
Human JAK2(WT/Full)	F: ATGGGAATGGCCTGCCTTACG<br>R: TCATCCAGCCATGTTATCCCTTA
Human JAK2(JH3-7)	F: ATGGGAATGGCCTGCCTTACG<br>R: CATTTGGTTCATATGAGTAGGCCT
Human JAK2(JH2)	F: GTGTTTCACAAAATCAGAAATG<br>R: CAAACTGTTAAGATCTCGTATGAT
Human JAK2(JH1)	F: TTGTTTACTCCAGATTATGAACTA<br>R: TCATCCAGCCATGTTATCCCTTA
Human JAK2(K882E)	F: GGAGGTGGTCGCTGTAGAAAAGCTTCAG<br>R: CTACAGCGACCACCTCCCCAGTGTTGT
