# Supplementary material for: IL-6 regulates autophagy and chemotherapy resistance by promoting BECN1 phosphorylation
Source: Nat Commun. 2021 Jun 15;12:3651. doi: 10.1038/s41467-021-23923-1 (PMC8206314; doi:10.1038/s41467-021-23923-1)
Supplement: Supplementary file 3 — Reporting Summary [file 41467_2021_23923_MOESM3_ESM.pdf]

nature research

Corresponding author(s): Guihua Wang

Last updated by author(s): 2021-4-22

Reporting Summary

Nature Research wishes to improve the reproducibility of the work that we publish. This form provides structure for consistency and transparency in reporting. For further information on Nature Research policies, see our Editorial Policies and the Editorial Policy Checklist.

Statistics

For all statistical analyses, confirm that the following items are present in the figure legend, table legend, main text, or Methods section.

n/a	Confirmed	
☐	☒	The exact sample size (n) for each experimental group/condition, given as a discrete number and unit of measurement
☐	☒	A statement on whether measurements were taken from distinct samples or whether the same sample was measured repeatedly
☐	☒	The statistical test(s) used AND whether they are one- or two-sided<br>*Only common tests should be described solely by name; describe more complex techniques in the Methods section.*
☐	☒	A description of all covariates tested
☐	☒	A description of any assumptions or corrections, such as tests of normality and adjustment for multiple comparisons
☐	☒	A full description of the statistical parameters including central tendency (e.g. means) or other basic estimates (e.g. regression coefficient) AND variation (e.g. standard deviation) or associated estimates of uncertainty (e.g. confidence intervals)
☐	☒	For null hypothesis testing, the test statistic (e.g. F, t, r) with confidence intervals, effect sizes, degrees of freedom and P value noted<br>*Give P values as exact values whenever suitable.*
☒	☐	For Bayesian analysis, information on the choice of priors and Markov chain Monte Carlo settings
☐	☒	For hierarchical and complex designs, identification of the appropriate level for tests and full reporting of outcomes
☒	☐	Estimates of effect sizes (e.g. Cohen's d, Pearson's r), indicating how they were calculated

Our web collection on statistics for biologists contains articles on many of the points above.

Software and code

Policy information about availability of computer code

Data collection	χ2-Test, Two-tailed Student's t-tests and Kaplan-Meier analysis were performed using Prism (version 8). Data for predicting the combination between JAK2 and BECN1 are accessible at String (https://string-db.org/). Data for the phosphorylation sites of BECN1 mediated by JAK2 are available in the multi-public datasets (KinasePhos2: http://kinasephos2.mbc.nctu.edu.tw/and Kinexus|PhosphoNet: http://www.phosphonet.ca/). Data for the molecular modeling of the interaction between BECN1-ECD and JAK2-JH1 are obtained from the PBD (https://www.rcsb.org/) and are modeled by MODELLER (https://salilab.org/modeller/), HDOCK (http://hdock.phys.hust.edu.cn/) and AMBER (http://ambermd.org/).
Data analysis	χ2-Test, Two-tailed Student's t-tests and Kaplan-Meier analysis were performed using Prism (version 8).

For manuscripts utilizing custom algorithms or software that are central to the research but not yet described in published literature, software must be made available to editors and reviewers. We strongly encourage code deposition in a community repository (e.g. GitHub). See the Nature Research guidelines for submitting code & software for further information.

Data

Policy information about availability of data

All manuscripts must include a data availability statement. This statement should provide the following information, where applicable:

- Accession codes, unique identifiers, or web links for publicly available datasets
- A list of figures that have associated raw data
- A description of any restrictions on data availability

Data for predicting the combination between JAK2 and BECN1 are accessible at String (https://string-db.org/). Data for the phosphorylation sites of BECN1 mediated by JAK2 are available in the multi-public datasets (KinasePhos2: http://kinasephos2.mbc.nctu.edu.tw/and Kinexus|PhosphoNet: http://www.phosphonet.ca/). Data

for the molecular modeling of the interaction between BECN1-ECD and JAK2-JH1 are obtained from the PBD (https://www.rcsb.org/) and are modeled by MODELLER (https://salilab.org/modeller/), HDOCK (http://hdock.phys.hust.edu.cn/) and AMBER (http://ambermd.org/).The data generated or analyzed during the current study are available within the article, supplementary information, and attached source data file or from the corresponding author upon reasonable request.

Field-specific reporting

Please select the one below that is the best fit for your research. If you are not sure, read the appropriate sections before making your selection.

☒ Life sciences ☐ Behavioural & social sciences ☐ Ecological, evolutionary & environmental sciences

For a reference copy of the document with all sections, see nature.com/documents/nr-reporting-summary-flat.pdf

Life sciences study design

All studies must disclose on these points even when the disclosure is negative.

Sample size	Samples size for each experiment is indicated in the figures or corresponding figure legends. The sample size was chosen based on previous experience in the lab, for each experiment to yield high power to detect specific effects.
Data exclusions	No data were excluded from the analysis.
Replication	All the experiments were performed in at least 2 biologically independent replicates. All replicates reported in the manuscript and on which statistics are based are biological replicates. No technical replicates were used to calculate statistics. All attempts at replication of the results were successful.
Randomization	Mice were randomized to behavioral group.
Blinding	Investigators were blinded to mouse genotypes during experiments, for performing sample analysis, imaging and quantification. For experiments with cell lines the researchers were blinded to cell line genotypes or treatment conditions for analysis, imaging and quantification.

Reporting for specific materials, systems and methods

We require information from authors about some types of materials, experimental systems and methods used in many studies. Here, indicate whether each material, system or method listed is relevant to your study. If you are not sure if a list item applies to your research, read the appropriate section before selecting a response.

Materials & experimental systems

n/a	Involved in the study	
☐	☒	Antibodies
☐	☒	Eukaryotic cell lines
☒	☐	Palaeontology and archaeology
☐	☒	Animals and other organisms
☒	☐	Human research participants
☒	☐	Clinical data
☒	☐	Dual use research of concern

Methods

n/a	Involved in the study	
☒	☐	ChIP-seq
☐	☒	Flow cytometry
☒	☐	MRI-based neuroimaging

Antibodies

Antibodies used	The antibodies used in here were as follows: Anti-MAP1LC3B (Cat#3868, Cell Signaling Technology, USA), Anti-GAPDH (Cat#sc-32233, Santa Cruz Biotechnology, USA), Anti-SQSTM1/p62 (Cat#66184-1-Ig, Proteintech Group, Wuhan), Anti-VPS34 (Cat#12452-1-AP, Proteintech Group, Wuhan), Anti-VPS15 (Cat# 17894-1-AP, Proteintech Group, Wuhan), Anti-Bcl-2 (Cat#15071, Cell Signaling Technology, USA), Anti-Rubicon (Cat#7151, Cell Signaling Technology, USA), Anti-ATG14 (Cat#96752, Cell Signaling Technology, USA), Anti-UVRAG (Cat#13115, Cell Signaling Technology, USA), Anti-STAT3 (Cat#9139, Cell Signaling Technology, USA), Anti-p-STAT3 (Y705) (Cat#9145, Cell Signaling Technology, USA), Anti-GFP (Cat#2955, Qi dongzi company, Wuhan), Anti-JAK1 (Cat#29261, Cell Signaling Technology, USA), Anti-p-JAK1 (Cat#74129, Cell Signaling Technology, USA), Anti-JAK2 (Cat#3230, Cell Signaling Technology, USA), Anti-p-JAK2 (Cat#ab32101, Abcam, USA), Anti-BECN1 (Cat#3495, Cell Signaling Technology, USA and Cat#66665-1-Ig, Proteintech Group, Wuhan), Anti-HA (Cat#3724, Cell Signaling Technology, USA, Anti-Flag (Cat#14793, Qi dongzi company, Wuhan), Anti-P-Tyr-100 (Cat#9411, Cell Signaling Technology, USA), Anti-Caspase3 (Cat#9662, Cell Signaling Technology, USA), Anti-Cleaved Caspase3 (Cat#9664, Cell Signaling Technology, USA), Anti-Ki-67 (Cat#9449, Cell Signaling Technology, USA), Anti-PARP (Cat#9532, Cell Signaling Technology, USA), Anti-Cleaved PARP (Cat#5625, Cell Signaling Technology, USA) and Anti-a-tublin (Cat#2148, Cell Signaling Technology, USA). p-BECN1 (Y333) specific antibody and the specific peptide for the site of BECN1 Y333 were made and purchased by Proteintech Group. The dilution used for each antibody was determined by the recommendations of the manufacturer.
Validation	The antibodies are commercially available and were validated by the provider. We used the protocols and recommendations of the

Validation	manufacturer only on validated species.

Eukaryotic cell lines

Policy information about cell lines

Cell line source(s)	The human cancer cell lines HCT116, LoVo, MCF-7, SW48 and the prostate cancer cell line PC3 and HEK293T cell lines, CT26 murine colon cancer cell were purchased from the American Type Culture Collection.
Authentication	None of the cell lines have been authenticated.
Mycoplasma contamination	The cell lines were not tested for mycoplasma contamination.
Commonly misidentified lines (See ICLAC register)	No

Animals and other organisms

Policy information about studies involving animals; ARRIVE guidelines recommended for reporting animal research

Laboratory animals	Balb/c female mice were bred in-house and injected subcutaneously with tumor cells after obtaining the approval by the institution's animal experimentation ethics committee of Huazhong university of science and technology, Tongji hospital. After injection one week, mice received intratumoral injection with IL-6 alone or in combination with CQ, and intraperitoneal injection with Oxaliplatin or twice a week. Tumor growth was measured by every other day measurements of tumor length (L) and width (W). The housing conditions of all animals were strictly following the ethical regulations. The room temperature ranged from 20 and 25°C. The relative ambient humidity at the level of mouse cages was 55 percent +/-15. Each cage was provided with food, water and two types of nesting material. Semi natural light cycle of 12:12 was used.
Wild animals	None
Field-collected samples	No field collected samples were used in the study.
Ethics oversight	The study was approval by the institution's ethics committee of Huazhong university of science and technology, Tongji hospital.

Note that full information on the approval of the study protocol must also be provided in the manuscript.

Flow Cytometry

Plots

Confirm that:

☒ The axis labels state the marker and fluorochrome used (e.g. CD4-FITC).

☒ The axis scales are clearly visible. Include numbers along axes only for bottom left plot of group (a 'group' is an analysis of identical markers).

☒ All plots are contour plots with outliers or pseudocolor plots.

☒ A numerical value for number of cells or percentage (with statistics) is provided.

Methodology

Sample preparation	The apoptosis analysis was performed using flow cytometry according to the instruction of Annexin V-PE/7-AAD Apoptosis Detection Kit. Cancer cells were seeded in 96-well plates and pretreated with IL-6 (20ng/mL) for 8 hours. Then, cells were cultured with chemotherapy drugs (5-Fu or OXA) alone or in combination with CQ (25 μl) for 36 hours. Cells were harvested in AnnexinV Binding Buffer without EDTA and stained with Annexin V-PE (5 μl) and 7-ADD (5 μl) for 30min in the dark at room temperature. Then, the cell apoptosis rate was measured by flow cytometry.
Instrument	BD FACSAria II (BD Biosciences)
Software	Raw FCS files were analyzed with FlowJo software (Treestar, Ashland, OR).
Cell population abundance	About 20, 000 single cells per sample were sorted and analyzed.
Gating strategy	Gating strategy to identify apoptotic cells with different treatments. Annexin V-PE and 7-ADD were used in the analyzed samples. Relative percentages for the gate are presented on the contour plot on the below, while event counts for each quadrant are presented on the four scatter plots on the above in supplement figure 6e.

☒ Tick this box to confirm that a figure exemplifying the gating strategy is provided in the Supplementary Information.
